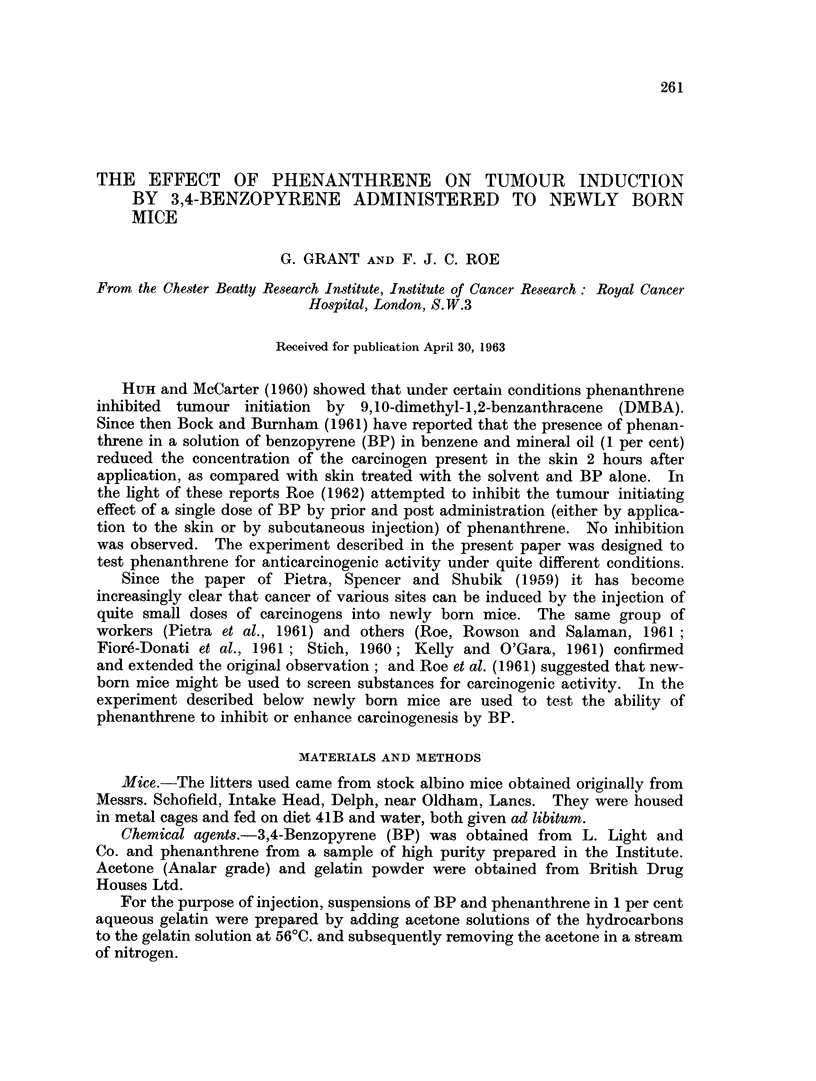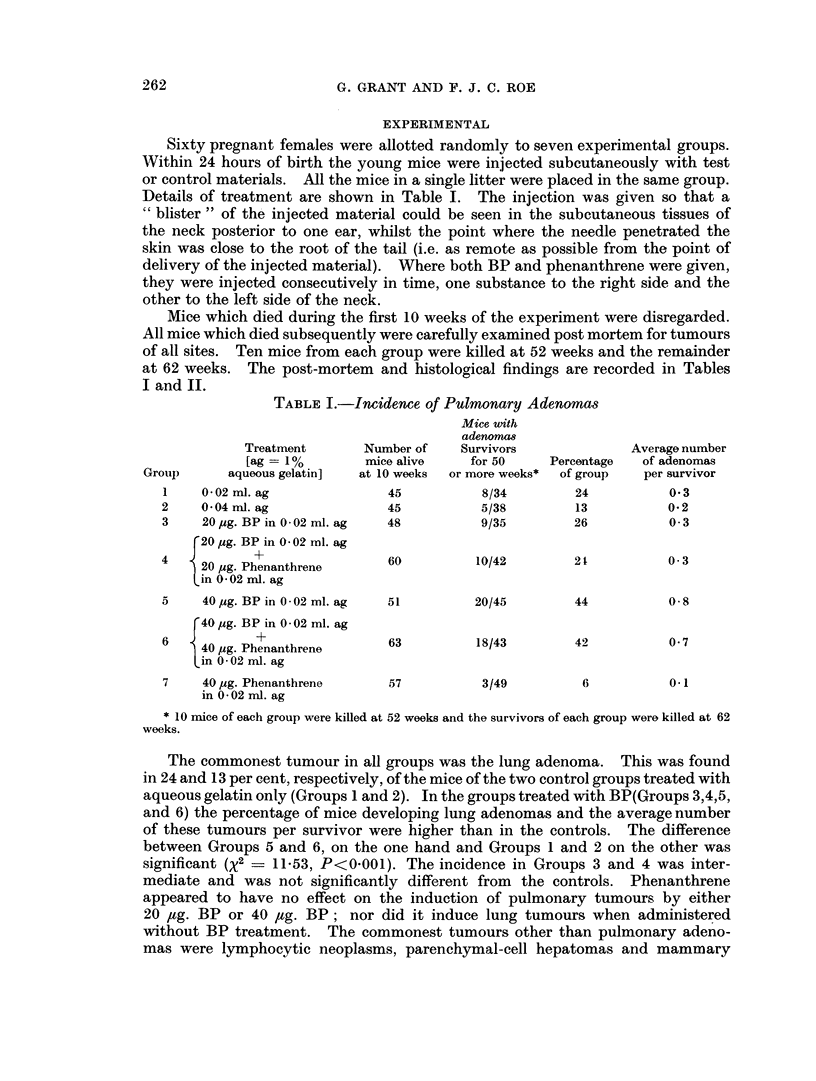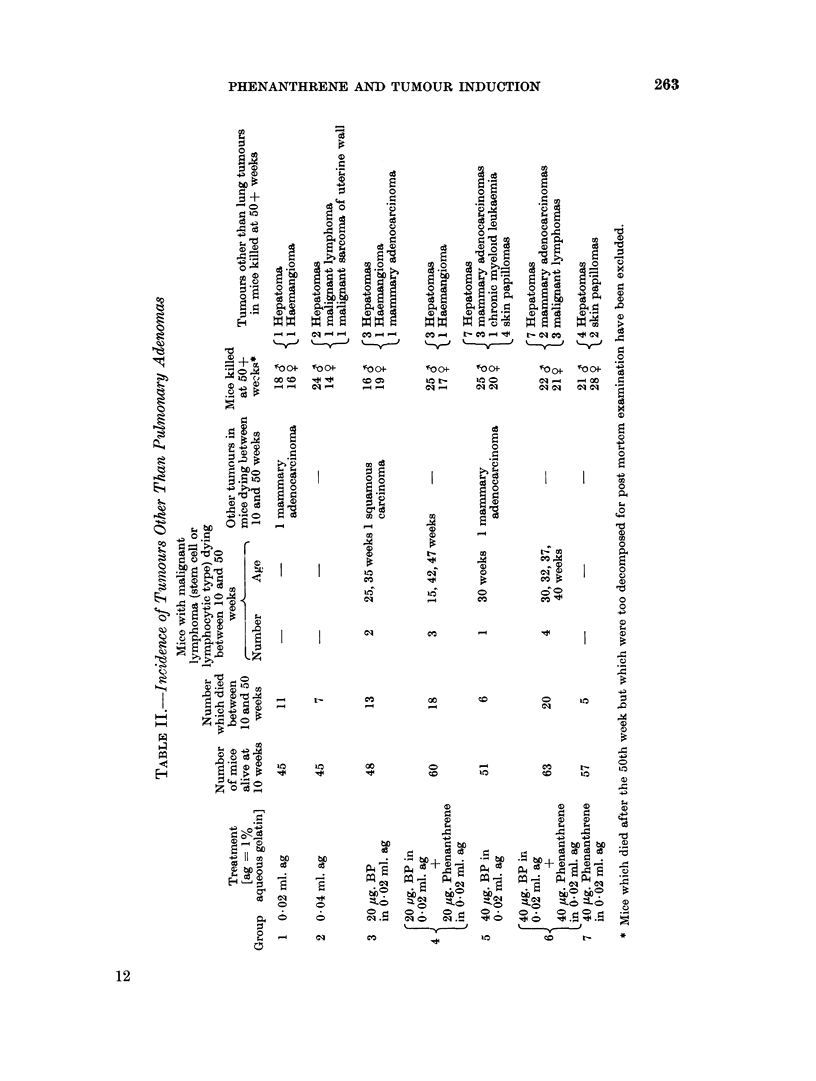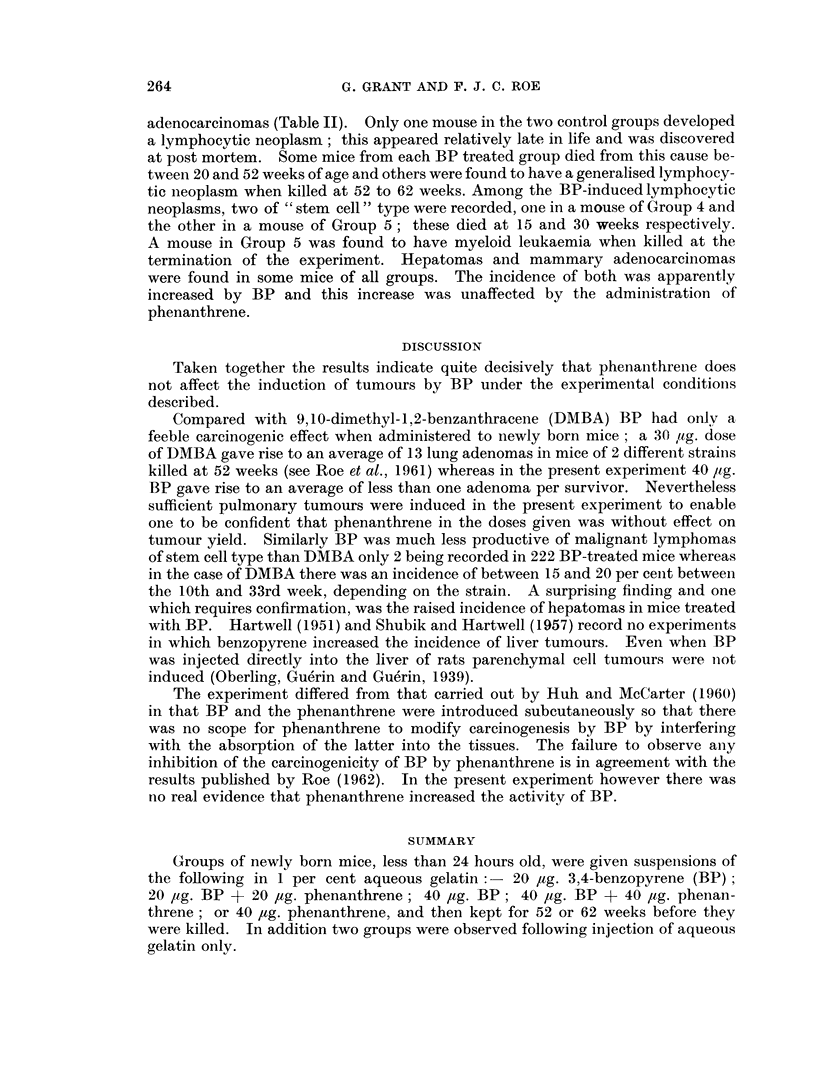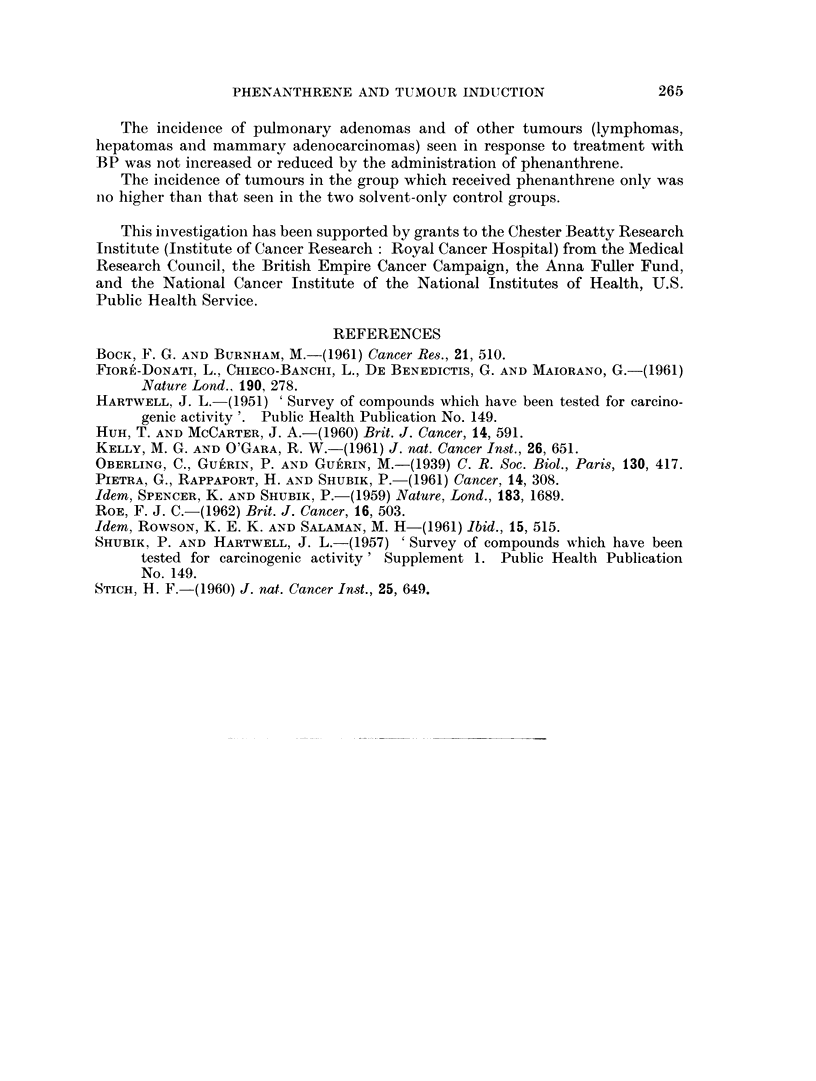# The Effect of Phenanthrene on Tumour Induction by 3,4-Benzopyrene Administered to Newly Born Mice

**DOI:** 10.1038/bjc.1963.37

**Published:** 1963-06

**Authors:** G. Grant, F. J. C. Roe


### THE EFFECT OF PHENANTHRENE ON TUMOUR INDUCTION BY 3,4-BENZOPYRENE ADMINISTERED TO NEWLY BORN MICE

G. GRANT AND F. J. C. ROE

*From the Chester Beatty Research Institute, Institute of Cancer Research : Royal Cancer Hospital, London, S.W.3*

Received for publication April 30, 1963

HUH and McCarter (1960) showed that under certain conditions phenanthrene inhibited tumour initiation by 9,10-dimethyl-1,2-benzanthracene (DMBA). Since then Bock and Burnham (1961) have reported that the presence of phenanthrene in a solution of benzopyrene (BP) in benzene and mineral oil (1 per cent) reduced the concentration of the carcinogen present in the skin 2 hours after application, as compared with skin treated with the solvent and BP alone. In the light of these reports Roe (1962) attempted to inhibit the tumour initiating effect of a single dose of BP by prior and post administration (either by application to the skin or by subcutaneous injection) of phenanthrene. No inhibition was observed. The experiment described in the present paper was designed to test phenanthrene for anticarcinogenic activity under quite different conditions.

Since the paper of Pietra, Spencer and Shubik (1959) it has become increasingly clear that cancer of various sites can be induced by the injection of quite small doses of carcinogens into newly born mice. The same group of workers (Pietra *et al.*, 1961) and others (Roe, Rowson and Salaman, 1961 ; Fioré-Donati *et al.*, 1961 ; Stich, 1960 ; Kelly and O'Gara, 1961) confirmed and extended the original observation ; and Roe *et al.* (1961) suggested that newborn mice might be used to screen substances for carcinogenic activity. In the experiment described below newly born mice are used to test the ability of phenanthrene to inhibit or enhance carcinogenesis by BP.

### MATERIALS AND METHODS

*Mice.*—The litters used came from stock albino mice obtained originally from Messrs. Schofield, Intake Head, Delph, near Oldham, Lancs. They were housed in metal cages and fed on diet 41B and water, both given *ad libitum.*

*Chemical agents.*—3,4-Benzopyrene (BP) was obtained from L. Light and Co. and phenanthrene from a sample of high purity prepared in the Institute. Acetone (Analar grade) and gelatin powder were obtained from British Drug Houses Ltd.

For the purpose of injection, suspensions of BP and phenanthrene in 1 per cent aqueous gelatin were prepared by adding acetone solutions of the hydrocarbons to the gelatin solution at 56°C. and subsequently removing the acetone in a stream of nitrogen.

### EXPERIMENTAL

Sixty pregnant females were allotted randomly to seven experimental groups. Within 24 hours of birth the young mice were injected subcutaneously with test or control materials. All the mice in a single litter were placed in the same group. Details of treatment are shown in Table I. The injection was given so that a "blister" of the injected material could be seen in the subcutaneous tissues of the neck posterior to one ear, whilst the point where the needle penetrated the skin was close to the root of the tail (i.e. as remote as possible from the point of delivery of the injected material). Where both BP and phenanthrene were given, they were injected consecutively in time, one substance to the right side and the other to the left side of the neck.

Mice which died during the first 10 weeks of the experiment were disregarded. All mice which died subsequently were carefully examined post mortem for tumours of all sites. Ten mice from each group were killed at 52 weeks and the remainder at 62 weeks. The post-mortem and histological findings are recorded in Tables I and II.

TABLE I.—*Incidence of Pulmonary Adenomas*

| Group | Treatment [ag = 1% aqueous gelatin] | Number of mice alive at 10 weeks | *Mice with adenomas* Survivors for 50 or more weeks* | Percentage of group | Average number of adenomas per survivor |
|---|---|---|---|---|---|
| 1 | 0·02 ml. ag | 45 | 8/34 | 24 | 0·3 |
| 2 | 0·04 ml. ag | 45 | 5/38 | 13 | 0·2 |
| 3 | 20 $\mu$g. BP in 0·02 ml. ag | 48 | 9/35 | 26 | 0·3 |
| 4 | 20 $\mu$g. BP in 0·02 ml. ag + 20 $\mu$g. Phenanthrene in 0·02 ml. ag | 60 | 10/42 | 24 | 0·3 |
| 5 | 40 $\mu$g. BP in 0·02 ml. ag | 51 | 20/45 | 44 | 0·8 |
| 6 | 40 $\mu$g. BP in 0·02 ml. ag + 40 $\mu$g. Phenanthrene in 0·02 ml. ag | 63 | 18/43 | 42 | 0·7 |
| 7 | 40 $\mu$g. Phenanthrene in 0·02 ml. ag | 57 | 3/49 | 6 | 0·1 |

* 10 mice of each group were killed at 52 weeks and the survivors of each group were killed at 62 weeks.

The commonest tumour in all groups was the lung adenoma. This was found in 24 and 13 per cent, respectively, of the mice of the two control groups treated with aqueous gelatin only (Groups 1 and 2). In the groups treated with BP(Groups 3,4,5, and 6) the percentage of mice developing lung adenomas and the average number of these tumours per survivor were higher than in the controls. The difference between Groups 5 and 6, on the one hand and Groups 1 and 2 on the other was significant ($\chi^2 = 11{\cdot}53$, $P < 0{\cdot}001$). The incidence in Groups 3 and 4 was intermediate and was not significantly different from the controls. Phenanthrene appeared to have no effect on the induction of pulmonary tumours by either 20 $\mu$g. BP or 40 $\mu$g. BP ; nor did it induce lung tumours when administered without BP treatment. The commonest tumours other than pulmonary adenomas were lymphocytic neoplasms, parenchymal-cell hepatomas and mammary

TABLE II.—*Incidence of Tumours Other Than Pulmonary Adenomas*

| Group | Treatment [ag = 1% aqueous gelatin] | Number of mice alive at 10 weeks | Number which died between 10 and 50 weeks | Mice with malignant lymphoma (stem cell or lymphocytic type) dying between 10 and 50 weeks: Number | Age | Other tumours in mice dying between 10 and 50 weeks | Mice killed at 50+ weeks* | Tumours other than lung tumours in mice killed at 50+ weeks |
|---|---|---|---|---|---|---|---|---|
| 1 | 0·02 ml. ag | 45 | 11 | — | — | 1 mammary adenocarcinoma | 18 ♂<br>16 ♀ | 1 Hepatoma<br>1 Haemangioma |
| 2 | 0·04 ml. ag | 45 | 7 | — | — | — | 24 ♂<br>14 ♀ | 2 Hepatomas<br>1 malignant lymphoma<br>1 malignant sarcoma of uterine wall |
| 3 | 20 $\mu$g. BP in 0·02 ml. ag | 48 | 13 | 2 | 25, 35 weeks | 1 squamous carcinoma | 16 ♂<br>19 ♀ | 3 Hepatomas<br>1 Haemangioma<br>1 mammary adenocarcinoma |
| 4 | 20 $\mu$g. BP in 0·02 ml. ag + 20 $\mu$g. Phenanthrene in 0·02 ml. ag | 60 | 18 | 3 | 15, 42, 47 weeks | — | 25 ♂<br>17 ♀ | 3 Hepatomas<br>1 Haemangioma |
| 5 | 40 $\mu$g. BP in 0·02 ml. ag | 51 | 6 | 1 | 30 weeks | 1 mammary adenocarcinoma | 25 ♂<br>20 ♀ | 7 Hepatomas<br>3 mammary adenocarcinomas<br>1 chronic myeloid leukaemia<br>4 skin papillomas |
| 6 | 40 $\mu$g. BP in 0·02 ml. ag + 40 $\mu$g. Phenanthrene in 0·02 ml. ag | 63 | 20 | 4 | 30, 32, 37, 40 weeks | — | 22 ♂<br>21 ♀ | 7 Hepatomas<br>2 mammary adenocarcinomas<br>3 malignant lymphomas |
| 7 | 40 $\mu$g. Phenanthrene in 0·02 ml. ag | 57 | 5 | — | — | — | 21 ♂<br>28 ♀ | 4 Hepatomas<br>2 skin papillomas |

* Mice which died after the 50th week but which were too decomposed for post mortem examination have been excluded.

adenocarcinomas (Table II). Only one mouse in the two control groups developed a lymphocytic neoplasm ; this appeared relatively late in life and was discovered at post mortem. Some mice from each BP treated group died from this cause between 20 and 52 weeks of age and others were found to have a generalised lymphocytic neoplasm when killed at 52 to 62 weeks. Among the BP-induced lymphocytic neoplasms, two of "stem cell" type were recorded, one in a mouse of Group 4 and the other in a mouse of Group 5 ; these died at 15 and 30 weeks respectively. A mouse in Group 5 was found to have myeloid leukaemia when killed at the termination of the experiment. Hepatomas and mammary adenocarcinomas were found in some mice of all groups. The incidence of both was apparently increased by BP and this increase was unaffected by the administration of phenanthrene.

### DISCUSSION

Taken together the results indicate quite decisively that phenanthrene does not affect the induction of tumours by BP under the experimental conditions described.

Compared with 9,10-dimethyl-1,2-benzanthracene (DMBA) BP had only a feeble carcinogenic effect when administered to newly born mice ; a 30 $\mu$g. dose of DMBA gave rise to an average of 13 lung adenomas in mice of 2 different strains killed at 52 weeks (see Roe *et al.*, 1961) whereas in the present experiment 40 $\mu$g. BP gave rise to an average of less than one adenoma per survivor. Nevertheless sufficient pulmonary tumours were induced in the present experiment to enable one to be confident that phenanthrene in the doses given was without effect on tumour yield. Similarly BP was much less productive of malignant lymphomas of stem cell type than DMBA only 2 being recorded in 222 BP-treated mice whereas in the case of DMBA there was an incidence of between 15 and 20 per cent between the 10th and 33rd week, depending on the strain. A surprising finding and one which requires confirmation, was the raised incidence of hepatomas in mice treated with BP. Hartwell (1951) and Shubik and Hartwell (1957) record no experiments in which benzopyrene increased the incidence of liver tumours. Even when BP was injected directly into the liver of rats parenchymal cell tumours were not induced (Oberling, Guérin and Guérin, 1939).

The experiment differed from that carried out by Huh and McCarter (1960) in that BP and the phenanthrene were introduced subcutaneously so that there was no scope for phenanthrene to modify carcinogenesis by BP by interfering with the absorption of the latter into the tissues. The failure to observe any inhibition of the carcinogenicity of BP by phenanthrene is in agreement with the results published by Roe (1962). In the present experiment however there was no real evidence that phenanthrene increased the activity of BP.

### SUMMARY

Groups of newly born mice, less than 24 hours old, were given suspensions of the following in 1 per cent aqueous gelatin :— 20 $\mu$g. 3,4-benzopyrene (BP) ; 20 $\mu$g. BP + 20 $\mu$g. phenanthrene ; 40 $\mu$g. BP ; 40 $\mu$g. BP + 40 $\mu$g. phenanthrene ; or 40 $\mu$g. phenanthrene, and then kept for 52 or 62 weeks before they were killed. In addition two groups were observed following injection of aqueous gelatin only.

The incidence of pulmonary adenomas and of other tumours (lymphomas, hepatomas and mammary adenocarcinomas) seen in response to treatment with BP was not increased or reduced by the administration of phenanthrene.

The incidence of tumours in the group which received phenanthrene only was no higher than that seen in the two solvent-only control groups.

This investigation has been supported by grants to the Chester Beatty Research Institute (Institute of Cancer Research : Royal Cancer Hospital) from the Medical Research Council, the British Empire Cancer Campaign, the Anna Fuller Fund, and the National Cancer Institute of the National Institutes of Health, U.S. Public Health Service.

### REFERENCES

Bock, F. G. and Burnham, M.—(1961) *Cancer Res.*, **21**, 510.

Fioré-Donati, L., Chieco-Banchi, L., De Benedictis, G. and Maiorano, G.—(1961) *Nature Lond.*, **190**, 278.

Hartwell, J. L.—(1951) ' Survey of compounds which have been tested for carcinogenic activity '. Public Health Publication No. 149.

Huh, T. and McCarter, J. A.—(1960) *Brit. J. Cancer*, **14**, 591.

Kelly, M. G. and O'Gara, R. W.—(1961) *J. nat. Cancer Inst.*, **26**, 651.

Oberling, C., Guérin, P. and Guérin, M.—(1939) *C. R. Soc. Biol., Paris*, **130**, 417.

Pietra, G., Rappaport, H. and Shubik, P.—(1961) *Cancer*, **14**, 308.

*Idem*, Spencer, K. and Shubik, P.—(1959) *Nature, Lond.*, **183**, 1689.

Roe, F. J. C.—(1962) *Brit. J. Cancer*, **16**, 503.

*Idem*, Rowson, K. E. K. and Salaman, M. H—(1961) *Ibid.*, **15**, 515.

Shubik, P. and Hartwell, J. L.—(1957) ' Survey of compounds which have been tested for carcinogenic activity ' Supplement 1. Public Health Publication No. 149.

Stich, H. F.—(1960) *J. nat. Cancer Inst.*, **25**, 649.